# Characterization of Transcription Factor Phenotypes within Antigen-Specific CD4+ T Cells Using Qualitative Multiplex Single-Cell RT-PCR

**DOI:** 10.1371/journal.pone.0074946

**Published:** 2013-10-04

**Authors:** Chansavath Phetsouphanh, Yin Xu, Janaki Amin, Nabila Seddiki, Francesco Procopio, Rafick Pierre Sekaly, John J. Zaunders, Anthony D. Kelleher

**Affiliations:** 1 Kirby Institute, University of New South Wales, Sydney, Australia; 2 Centre for Applied Medical Research, St Vincent's Hospital, Sydney, Australia; 3 Vaccine and Gene Therapy Institute (VGTI), Port St. Lucie, Florida, United States of America; New York University, United States of America

## Abstract

Current research on antigen specific CD4+ T cells indicates that there is functional and phenotypic heterogeneity within these populations, but the extent of this heterogeneity is poorly described. The CD134/CD25 assay allows live isolation of antigen specific cells *in vitro* for down-stream molecular analysis. Antigen specific CD4+ T cells were examined at the molecular level by lineage specific transcription factor profiling using qualitative multiplex single cell RT-PCR and Lock Nucleic Acid (LNA) probes allowed unbiased amplification and delineation of expression of *Tbx21*, *Gata3, Rorc, Foxp3* and *Bcl-6*. It overcomes the limitations of previous assays by allowing identification of transcription factor mRNA in single antigen specific cells with high sensitivity (down to 10 femtograms) and specificity. Patterns of responses can be robustly characterized using <200 cells based on exact binomial calculations. These results are reproducible with a CV of ≈6%. The patterns of heterogeneity are stable within an individual antigen specific response but vary between responses to different antigens. Responses to CMV have a Th1 predominant profile (35.6% of responding cells expressing *tbx21*) whereas responses to Tetanus Toxoid have a Th2 biased profile (22% of responding cells expressing *gata3*), with unexpectedly high levels of Treg cells found in both populations. Here we describe a methodology that allows live isolation of Ag specific cells and transcription factor profiling at a single cell level to robustly delineate the different CD4+ T cell subsets within this population. This novel method is a powerful tool that can be used to study CD4+ T cell heterogeneity within extremely small populations of cells and where cell numbers are limited.

## Introduction

A substantial limitation of studying antigen (Ag) specific T cell responses is that the standard approaches, such as ELISPOT and intra cellular cytokine (ICC) assays, do not simultaneously examine the responses of all CD4+ T cell types (Th1, Th2, Th17, Tfh and Tregs) [1]. Both approaches are limited by technical difficulties in multiplexing detection of cytokines and cell surface phenotypes (particularly ELISPOT), differences in time courses of cytokine production and the lack of good antibodies to a number of important cytokines, such as IL-10 and TGF-β [2], [3]. The outcome and effectiveness of a response may depend on the relative proportions of the different functional subsets that develop during the response to an Ag. Certainly this appears to be the case in some infections, such as in leprosy where the relative Th1 and Th2 responses appear to determine outcome [4]. However even in this model, relative responses across all functional subsets have not been documented [5]. An alternative approach of identifying antigen specific CD4+ T cells is based on simultaneous cell surface co-expression of 2 important activation markers, CD134 (Ox40) and CD25 (IL-2Rα) [6].

The CD134/CD25 assay is a novel technique that assesses Ag specific CD4+ T cell responses from whole blood or peripheral blood mononuclear cells (PBMC). Ag Specific CD4+ T cells can be identified in cultures stimulated with a range of Ags (i.e. peptides, proteins, lysates, inactivated pathogens or toxins) or mitogens, and subsequent measurement of combined cell surface expression of CD25 (IL-2Rα) and CD134 (OX40) just prior to 48hrs. Murine studies have demonstrated that OX40-OX40L interactions are crucial for generation of memory T cells. OX40 expression peaks 24–48 hrs after T cell receptor engagement. Surface co-expression of these important molecules provides a sensitive and specific means to identify and isolate human Ag specific CD4+ T cells for functional or molecular characterization [6]. Further, as CD134 and CD25 expression is up regulated on Th1, Th2, Th17, Tfh and Treg cells, this assay is more likely to capture the total or global CD4+ T cell response to an antigen rather than sampling an aspect of this response by measuring certain cytokines. Although responding cells can be enumerated, the assay cannot delineate the heterogeneity within this global response.

Research on Ag specific CD4+ T cells has been primarily done on bulk-sorted cells that are hypothesized to be a homogenous population. However, accumulated data demonstrate that there is heterogeneity within the Ag specific population on the basis of surface marker expression and cytokine secretion profile. These parameters may be regulated by the type of Ag encountered, mode of Ag exposure and Ag load [7]. One approach to understanding the level of heterogeneity within this population is investigation of individual cells at the molecular level. Lineage specific transcription factor (TF) profiling (Tbet (*Tbx21*) for Th1, *Gata3* for Th2, *Rorc* for Th17, *Bcl-6* for Tfh, *Foxp3* for Tregs and *Stats 1–6* for each subset) at the single cell level using RT-PCR together with the Ag specific OX40/CD25 assay is likely to give insight into the overall heterogeneity in the responses of CD4 T cells to an antigen.

Expression analysis using *in vitro* protein assays on individual cells is generally insufficiently sensitive, with the exception being FACS (Fluorescence Activated Cell Sorting), which has, revealed diversity of cellular populations that previously appeared superficially similar. A more sensitive methodology with which to study the transcriptional networks within a given population is by measuring gene expression at a single cell level [8]. Reverse Transcription-Polymerase Chain Reaction (RT-PCR) allows the detection of rare RNA messages from individual cells, possibly down to a few copies per cell. The main obstacle of this is the quantity of RNA recoverable from a single cell. A single cell contains approximately 10–40 pg of RNA, of which 0.1–10 pg is mRNA (∼10,000 genes), which corresponds to 10^5^ to 10^6^ message copies [9], [10]. Thus, there are several difficulties in developing a methodology that will enable multiple mRNA detection within a single cell. Factors such as competition between multiple primers, linearity of reverse transcription and pre-amplification steps, sensitivity and reproducibility of the assay must be taken into account [11].

Profiling of mRNA in single cells quantitatively has revealed that within phenotypically similar cells, heterogeneity of mRNA transcript levels is substantial [12]–[14]. Here we have developed a methodology that allows live isolation of Ag specific cells that enables transcription factor profiling at a single cell level to delineate the different CD4+ T cell subsets within this population. This assay overcomes the limitations of previous assays by allowing identification of single Ag specific cells with improved sensitivity and specificity. It reveals that there is in fact substantial heterogeneity within antigen specific responses and that these patterns vary by antigen response. This novel method is a powerful tool that can be used to study heterogeneity within extremely small populations of cells and lead to better understanding of the determinate to outcomes of antigen specific responses.

## Methods

### Ox40/CD25 Assay

PBMC were cultured in 0.5 ml of IMDM (JRH) with 10% Human AB serum (Lonza) in 24-well plates (BD Biosciences). Individual cultures contained CMV lysate (grade III; Meridian Life Science) at a final concentration of 2 µg/ml; Tetanus Toxoid (Commonwealth Serum Laboratories, Melbourne, Australia) at a final concentration of 2lfU/ml); or HIV Clade B gag pool of 123 15mer overlapping peptides (NIH AIDS Research and Reference Reagent Program) used at a final concentration of 2 µg/ml for each peptide. Cultures were incubated at 37°C for 48 hrs in a humidified atmosphere of 5% CO2 in air. Negative control cultures comprised PBMCs mixed with IMDM with 10% AB serum only while SEB was used for positive control cultures [6] [15].

### Ethics statement

PBMCs were extracted from whole blood from healthy volunteers for the purpose of development, optimization and validation of the assays using Ficoll density centrifugation. The volunteers gave written informed consent. The St Vincent's Research Ethics Committee approval number was EC00140.

### Single cell sorting

After 48 hrs the culture was stained extracellular with CD3- PerCP-Cy5.5, CD4-PE-Cy7, CD25APC, and CD134-PE (BD Biosciences) for 20 min, washed with PBS (GIBCO, Invitrogen) at 1200 rpm for 7 mins. The supernatant was decanted and the cells re-suspended in 500 ul PBS with 10% FCS (Boyogen Biologicals). The suspension was then filtered with a 70 μm filter, ready for sorting on the FACS Aria (BD). FACS sorting is a crucial step in this assay. Ensuring that a single cell is dispensed appropriately into individual wells is critical. Stringent conditions such as, drop delay, doublet exclusion, appropriate gating and droplet positioning were considered to enable high sort efficiencies. To ensure that the Aria consistently sorts 1 cell per well, 500 PBMC were sorted directly onto a haemo-cytometer slide and then 1 HUT78-GFP+ cell was sorted on top. The slide was then imaged on a fluorescence microscope. Normal light was used to focus on the HUT78 with surrounding PBMC and then the FITC channel was used to detect Green Fluorescent Protein expressing cells. Only the GFP expressing HUT78 cell fluoresces in this channel, confirming that the ARIA was sorting 1 cell per well (Fig. S1a). This was repeated 5 times to ensure reproducibility. HUT78 cells were provided by NIH AIDS Research & Reference Reagent Program (Catalogue #89).

The Ag specific CD25+CD134+ CD4+ T cell population was sorted into a fully skirted 96 well plate (Eppendorf), 1 cell per well. The 96 well plates had 5 μl of 2× mix (buffer containing 0.4 mM of each dNTP and 3.2 mM MgSO4) (Invitrogen), 1.8 μl of PCR grade water (GIBCO, Invitrogen) and 0.1 μl Superase In (Promega). The plates were then stored at −80°C.

### Primer design and *in silico* dynamics

Primers for all TF were designed using the ROCHE Universal Probe Library® **ProbeFinder** assay design centre. The design centre software is based on **Primer3** software, with strict parameters that account for primer size, Tm, Self- dimer and GC content [16]. All primer pairs designed using the **ProbeFinder** were checked with the *in silico* algorithm that examines the entire genome and transcriptome for any possible mispriming sites, which it filters. The primers were then further scrutinized by a primer BLAST (NCBI) search to ensure that they are within the right genes and to identify any mis-matches. Integrated DNA technologies (IDT) analysis tool was then used to determine primer kinetics and as the assay is multiplex, the tool was used to scrutinize for secondary structure (hairpin), self-dimer, hetero-dimer formation and to confirm that they were intron-spanning. Primers ranged from 18–28 bp with approximately 50% GC content and Tm <60°C. Secondary structure or Hairpins were excluded if their ΔG is lower than −3 kcal/mol and Tm >50°C. Self–dimer is the ability of the primer to bind in a homologous way to itself. ΔG of less than −6 kcal/mol is excluded, unless there is no indication of primer-dimer with ΔGs between −10 kcal/mol to −6 kcal/mol. Hetero-dimer examines intermolecular interactions between 5′ sense and 3′ non-sense primers (ΔG of greater than −6 kcal/mol is tolerated). Hetero-dimer can also be measured between the primer and its template (target), the lower the ΔG, the less energy required for spontaneous interaction (Table. S1). To achieve full confidence in the primer sets, each primer must have met all the above-mentioned parameters and complied with the MIQE guidelines [17].

### 1^st^ round RT and multiplex pre-amplification

Sorted 96 well plates were thawed at room temperature; 3 μl of primer mix (containing 40 nM *Foxp3*, 30 nM *Tbx21/Rorc/Gata3*, 25 nM *bcl-6/ß-actin and STATs*) with 0.2 μl Superscript III/*Taq* polymerase (Invitrogen) was then added to the wells. The 1^st^ round RT and pre-amplification was done on an Eppendorf thermocycler, the cycling conditions were 51°C for 15 min, 95°C for 2 min then 22 cycles of 95°C for 15 sec 60°C for 4 min. The 1^st^ round products were then diluted 1 in 5 by adding 40 ul of 1× TE (at this step the plate can be stored at −80°C prior to 2^nd^ rd detection).

### 2^nd^ round real-time PCR

2 μl aliquots of the 1^st^ round product were placed into individual wells in a 384 well plate (ROCHE). 8 μl of master mix was then added to the wells (containing 5 μl of Probe Master (ROCHE), 0.5 μl of Forward primer, 0.5 μl Reverse primer, 0.25 μl UPL Probe (ROCHE) and 1.75 μl of PCR grade water (ROCHE)). The 384 well plate was then placed in a ROCHE Lightcyler 480® for real-time detection, where the cycling conditions were 95°C for 2 min and 45 cycles of 95°C for 10 sec, 60°C for 30 sec, 72°C for 1 sec. Analysis of real-time data was done using Light-cycler software.

### CD4 T cell activation and RNA extraction

Positive RNA controls for the scRT-PCR assay were made using purified CD4+ T cells, which had been stimulated *in vitro* with 250 ng/ml of PMA and 1 ug/ml of Ionomycin, for 4 hr. The cells were then lysed with 0.75 ml TRIZOL (Invitrogen) and 200 μl of chloroform (Sigma) was then added. The mix was then centrifuged at 12000 g for 15 min at 4°C. The clear aqueous phase was transferred to a clean 1.5 ml tube (Eppendorf). 2 μl of Glycogen (ROCHE) and ∼600 μl of iso-propanol (Sigma) added and then centrifuged at 12000 g for 15 min at 4°C, supernatant aspirated and the pellet washed with ice cold 70% ethanol (Sigma) and supernatant discarded after centrifugation again at 7500 g. The resulting pellet was air dried, the RNA re-suspended in 20 μl DEPC DDW (Promega), and stored at −80°C.

### Statistical analyses

Prism 5.0a (GraphicPad) was used for analyses of linear regression and exact binomial calculations. Wilcoxon paired *t* test was used to analyse statistical data on the Prism 5.0a (GraphicPad) software. *p* values <0.05 were considered significant.

## Results

### Overview of the Qualitative Multiplex single cell RT-PCR assay (scRT-PCR)

**Figure 1:**
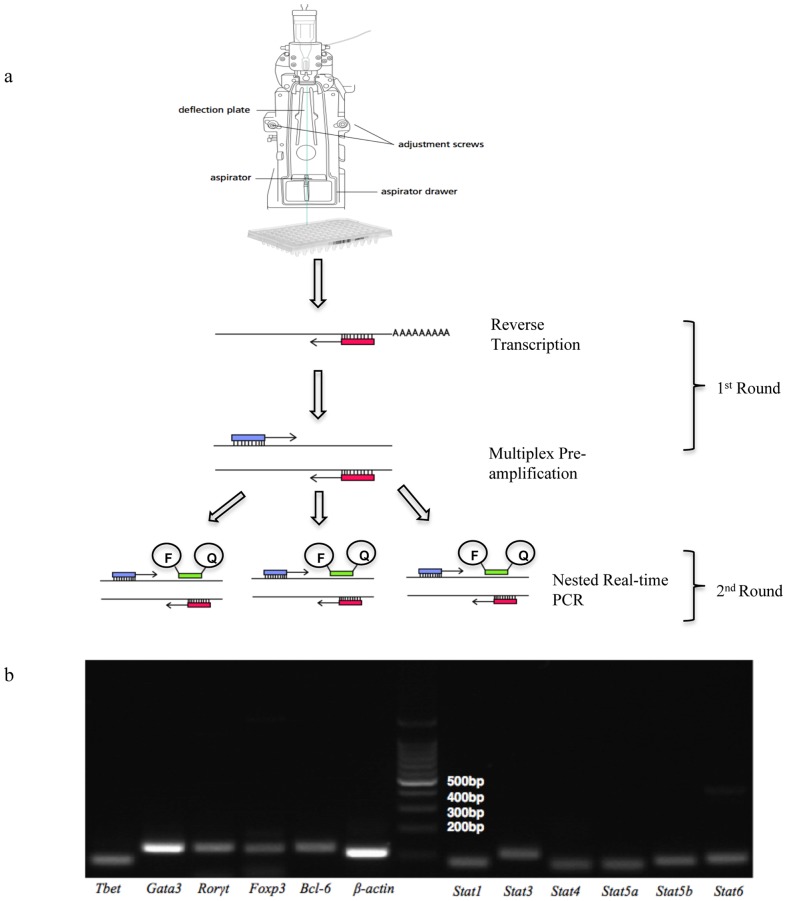
Overview of the qualitative multiplex single-cell RT-PCR and Primer Specificity. A) Single cells are sorted into individual wells in a 96 well plate. Binding of the gene specific 3′ primer to the mRNA allows reversed transcription to cDNA. This is then followed by 22 cycles of multiplex pre-amplification. The 1^st^ round products are diluted 1/5 and then split for the 2^nd^ round real-time amplification with nested 5′ and 3′ primers, and specific amplification was detected with the addition of specific LNA probes (F = FAM, Q = Quencher). B) Specificity of all 11 TF and *ß-actin* (positive signal control) was examined by electrophoresis in 1.5% Agarose gel, to determine correct sized amplicons (for amplicon sizes refer to Supplementary). Real-time PCR was subsequently used to confirm specificity by checking single melt peaks using SYBR®-green I.

Single cells were sorted by FACSAria into individual wells in a 96 well PCR plate containing 2× reaction buffer (Mg^2+^, dNTPs and stabilizers) and RNAse inhibitor, the plates were then frozen and stored at −80°C. Before 1^st^ round RT pre-amplification, 12 primer pairs and Superscript III/Taq polymerase enzymes were added. Lysis of the cells was achieved by differential osmolality between the cells and the PCR buffer and heating to 51°C prior to the RT step. This process allowed accessibility of cellular contents, namely mRNA, for the gene specific 3′ reverse primers and superscript III reverse transcriptase to bind for cDNA synthesis (Fig. 1a). 22 cycles of multiplex pre-amplification was necessary to ensure that the low level mRNA transcripts within each cell were detectable in real-time PCR, as *ß-actin* expression was observed at ∼24 C_q_ in the 2^nd^ round real-time step, this ensures that the high copy mRNA detection is not biased within the assay and the assay also remains linear (Fig. S2). The 1^st^ round product was diluted and split into aliquots for 2^nd^ round nested real-time PCR in a 384 well plate for subsequent detection of each individual transcription factor.

### Assay Validation

#### Specificity

To ensure correct amplification of each TF, all primers were tested for specificity (Fig. 1b). Firstly, each primer set produced the expected sized amplicon as demonstrated on 1.5% Agarose gel. Before the use of Locked Nuclei Acid (LNA) probes, the primer specific amplicons were examined using SYBR®-green I based real-time PCR allowing confirmation of a single peak in the melt curve for each product. Further, each amplicon was sequenced to ensure specific amplification (data not shown). UPL® LNA probes are short 8–9 bp probes that contain DNA nucleotide analogues (LNA), a FAM labeled 5′ end and a 3′ end quencher. These probes have increased binding strength compared to standard DNA nucleotides, enhancing specificity of the PCR, and eliminating non-specific fluorescence detected when using SYBR®-green I dyes [18].

### Reverse Transcription

**Figure 2:**
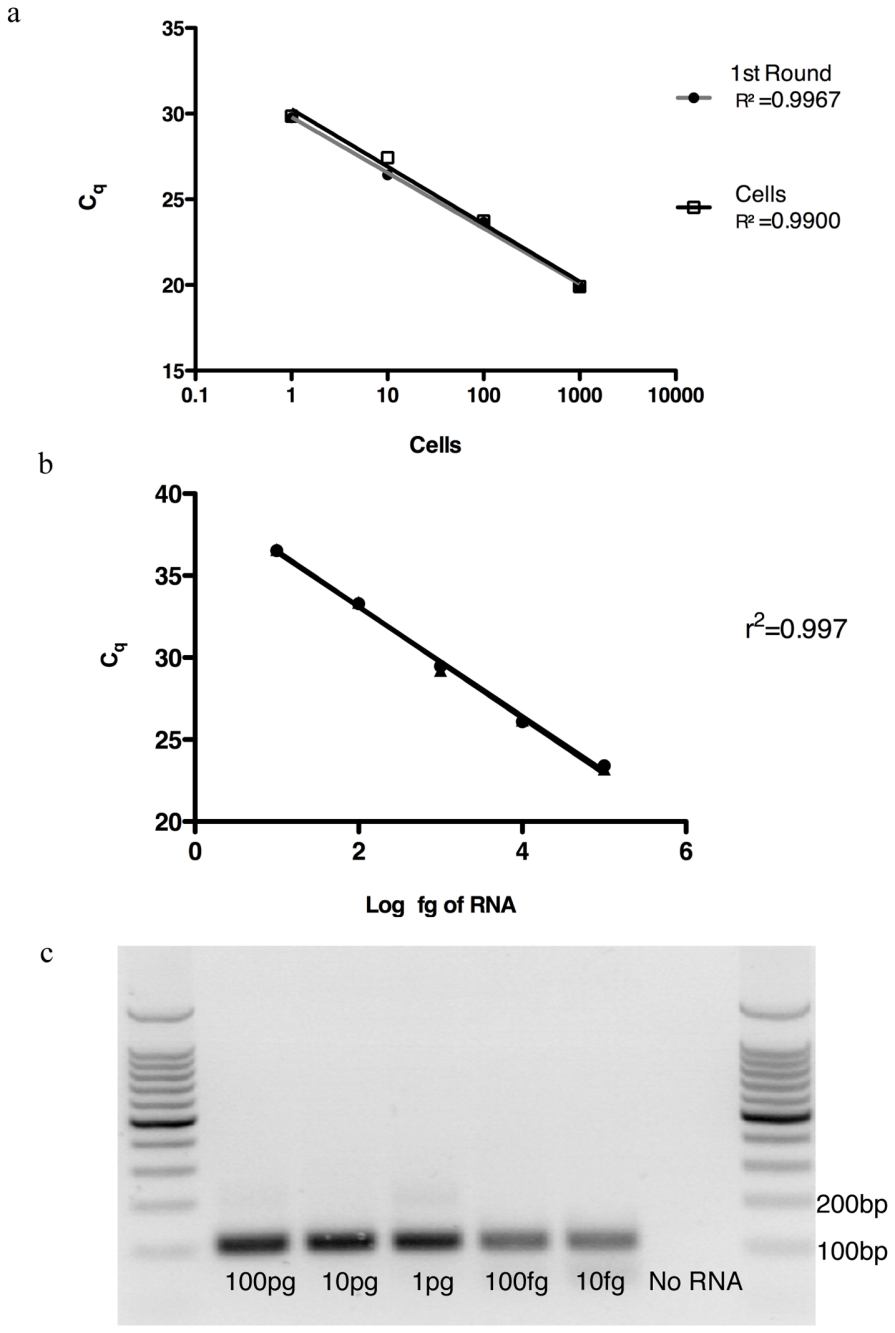
Assessment of reverse transcription efficiency and assay sensitivity. A) CD25+ CD134+ CD4 T cells were sorted 1000, 100, 10 and 1 cell. All cell number was reverse transcribed under the same conditions. The 1^st^ round product (filled circle) of 1000 cells was then diluted to the appropriated concentrations corresponding to the cell numbers. 2^nd^ round real-time amplification of *ß-actin* was then performed. A) The linear regression for the 1st round products and the cells (empty squares) were r^2^ = 0.9967; Slope =  −3.24, r^2^ = 0.9900; Slope =  −3.34 respectively. All replicates were performed in triplicate. B) Varying amounts of RNA from activated CD25+CD134+ CD4 cells were used in the multiplex single-cell RT-PCR assay from 100 pg to 10 fg. *ß-actin* expression (duplicate) is detectable at all levels and the reverse transcription of the assay is linear with r^2^ = 0.997. C) Representative gel from the same PCR run, with *ß-actin* specific band at 100bp. All replicates were performed in triplicate.

Reverse transcription (RT) is a pivotal step up-stream of multiplex pre-amplification and real-time detection, as it may introduce biases based on the secondary and tertiary structures of the mRNA, variation in priming efficiency and discrimination of low mRNA copies [19]. All of these factors depend on the efficiency of the reverse transcriptase used. In order to address these issues, we sorted predetermined numbers of CD4+ T cells: 1000, 100, 10 and 1 cell. Each aliquot underwent the same reverse transcription and pre-amplification conditions (as described in methods). The 1^st^ round product from 1000 cells was then serial diluted to equivalent concentrations (1/10, 1/100, 1/1000); and then used for *ß-actin* detection in the 2^nd^ round real-time PCR (Fig. 2a). Linear regression analysis demonstrated that the results of the 1^st^ round products correlated very strongly with the results from individual cell numbers (r^2^: 0.9967 and 0.9900 respectively), an indication of good RT efficiency. It also indicated that the RT reaction was linear; whereby 10 fold differences in mRNA copies differ by 3 cycles. Potential variation in priming of the reverse transcriptase was negated by using gene specific primers, allowing specific RT of desired genes as opposed to general RT of the transcriptome using random hexamers or oligo-dT.

### Primer Competition

**Figure 3:**
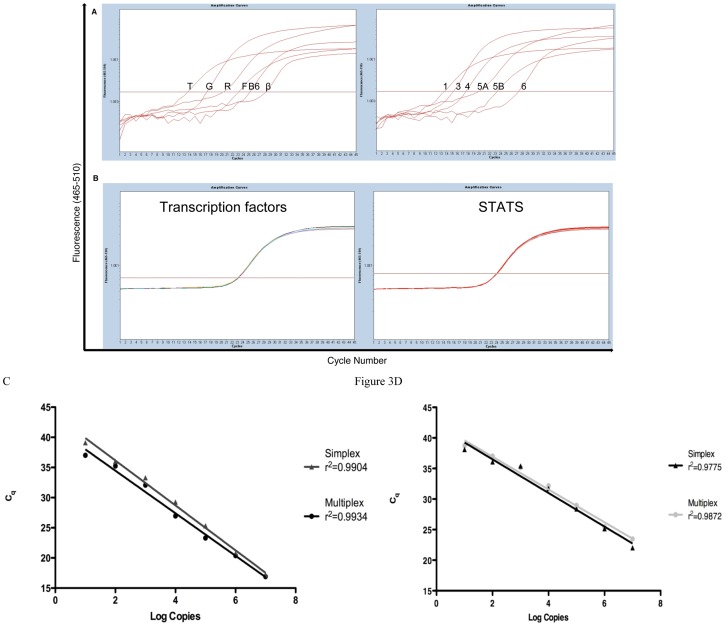
Primer competition in the multiplex pre-amplification PCR and Linearity/Dynamic range of the assay. A) Known concentrations of *Tbx21 (T), Gata3 (G), Rorγt (R), Foxp3 (F), Bcl-6 (B6)*, and *ß-actin (B)* DNA products (1, 1/10,1/100, 1/1000, 1/10000 and 1/100000 respectively), as well as, *stat1, 3, 4 5A, 5B and stat6* (1, 1/10,1/100, 1/1000, 1/10000 and 1/100000 respectively) were mixed in the 1^st^ round RT-PCR step. The different ratios of each product detected, reflect the initial dilutions of the different products, C_q_ were ∼2.8 cycles apart. The same concentration of 1/1000 was used for the all products; the C_q_ was ∼22.5 with a CV of 0.3%. C) *ß-actin* plasmid standards from 10^7^ to 10 copies were used in the 2 step multiplex assay and a simplex real-time PCR assay. The r^2^ values for the simplex (triangle) and the multiplex (circle) reaction are r^2^ 0.9904 and r^2^ 0.9934 respectively. D) *Bcl-6* plasmid standards from 10^7^ to 10 copies were used in the 2 step multiplex assay and a simplex real-time PCR assay. The r^2^ values for the simplex (triangle) and the multiplex (circle) reaction are r^2^ 0.9755 and r^2^ 0.9872 respectively. All replicates were performed in triplicate.

Primer competition is a major potential problem associated with multiplex PCR. In our case 24 primers (Table S1) were present within each reaction. Competition between primers for their targets may result in inhibition of amplification, which reduces PCR efficiency. Our primers were designed to amplify cDNA of comparable composition and length (78–130 bp). The small length of the amplicons limits reagent usage in the pre-amplification step and favors unbiased amplification based on abundance [11]. To address primer competition, we purified and quantified individual PCR products for each gene. These DNA products were then amplified in 1^st^ round pre-amplification (excluding the RT step) in combination with varying quantities of other products. Aliquots of the 1^st^ round products were then amplified in the 2^nd^ round for real-time detection. The conditions for all reactions were initially optimized with primers at 25 nM final, however when taking competition into consideration, optimal primer concentrations differed and were asymmetric, based on the annealing strength of each primer for their targets (see methods). To examine this directly, we used *Tbx21, Gata3, Rorγt, Foxp3, Bcl-6*, and *ß-actin* DNA products mixed at known concentrations (1, 1/10,1/100, 1/1000, 1/10000 and 1/100000). This was also done for *Stat1, 3, 4 5A, 5B and Stat6*. The different ratios of each product detected reflect the initial dilutions of the different products, as C_q_ were ∼2.8 cycles apart (Figure 3a). When the same concentration (1/1000) was used for all products; the C_q_ was the same for all three ∼22.5 with a CV of 0.3% (Figure 3b). This highlighted that by adjusting different primer concentrations based on their binding strengths can alleviate the problem of competition. It is also possible to simultaneously amplify multiple genes within the one reaction without biasing highly abundant transcripts and avoids discrimination of lower copy transcripts.

### Sensitivity

As a single cell contains 10 to 40 pg of RNA, of which ∼0.1–1 pg is mRNA with much of this representing low copy genes, it is vital that the assay is sensitive enough to detect mRNA at low levels [9]. To determine the sensitivity of the assay, elucidation of the RNA input amount is necessary (Fig. 2b&c). RNA extracts from PMA/Ionomycin activated CD4+ T cells were used as input template at varying concentrations (100 pg, 10 pg, 1 pg, 100 fg and 10 fg). *ß-actin* expression was detected at all concentrations with a linear regression coefficient of 0.997. The scRT-PCR assay is sensitive down to 10 fg, however at lower concentrations there would be excess primers as there is lower amounts of template for amplification, this in turn will lower the PCR efficiency in the 2^nd^ Round. To further test the sensitivity of the assay and examine the level of TF mRNA copies present in activated CD4+ T cells, total RNA extracted from PMA/Ionomycin activated CD4+ T cells were titrated from 100 pg to 10 fg. Most of the TF were detectable down to 10 fg, except for *Roryt*, which was present at slightly lower in copy numbers but was still detectable at 100 fg of total cellular RNA (Fig. S1b). The scRT-PCR assay has the capability of amplifying low copy mRNA, with assay sensitivity down to 10 fg of input RNA.

### Linear Dynamic Range

Although the initial goal was to design a qualitative assay for single-cell gene expression, the dynamic range and linearity of the assay was investigated in order to determine if quantitative mRNA expression within a single-cell could be achieved. The larger the dynamic range, the greater the ability to detect samples with high and low copy number in the same run [20]. We made DNA controls using PCR products and cloned them into plasmid vectors, which were quantified and then diluted 10 fold from 10^7^ to 10 copies. Normal distribution of template below 10 copies was not expected and if detection of lower copies is necessary Poisson distribution needs to be considered. This scRT-PCR assay however contains a pre-amplification step in which low mRNA copies are amplified ∼2^21^ times and therefore the genes of interest should be detectable within the dynamic range of the assay. To show linearity of the assay, plasmid standards were used in the 2 step multiplex assay and correlated with a simplex one-step real-time PCR (Fig. 3c&d). *ß-actin* plasmid standards from 10^7^ to 10 copies were used; the results for the simplex and the multiplex reaction were similar with r^2^ values of 0.990 and 0.993 respectively, showing that the assay can amplify linearly. Similar results were obtained with *Bcl-6* plasmid standards (r^2^simplex: 0.9755, multiplex: 0.9872) further confirming the linearity of the 2 step multiplex assay with the low end of the dynamic range being 10 copies.

### Precision of the Assay

To determine inter-assay (plate to plate) variation of the assay and to elucidate the number of cells required for reliable and precise results, 4 healthy controls that had known CD4+ T cell responses to CMV were used in the multiplex scRT-PCR assay (186 CD134+ CD25+ CD4+ T cells were sorted into individual wells for each patient). *Tbx21* expression in responding cells from the 3 plates was used to determine inter-assay variation. The co-efficient of variation (CV) averaged across the 4 samples (3 plates each; 62 cells per plate) was 6.05%, which reflects good inter-assay variation as each of the sets of plates contained non-identical single cells that were sorted from the same population. By using exact binomial proportion calculations with 95% CI we were able to determine the reproducibility and precision of the assay. As the estimated average width of the plate is reduced the reported results become more precise. By analyzing 3 plates per sample (186 cells) we were able to increase precision by 57% (from 1 plate average width of 24.83 to 3 plate average width of 14.18) in the case of Tbet (*tbx21)* expressing cells (Table 1). The improvement in precision was also evident for the other TF: *Gata3* (55.6%), *Rorc* (50.4%), *Foxp3 (56.9%)* and *Bcl-6* (53.4%) (Data not shown). By analyzing 3 plates per sample, it was possible to increase the precision of the assay by ∼54.7% and enables the determination of plate to plate variation with the sample as well as elucidating the minimum cell number required for the assay, in this case 186 cells. Similar to CMV specific responses Tetanus toxoid specific CD4 T cells expressing *gata3*, had an increased of 57.6% in precision, when analyzing 186 cells compared to 62 cells (Table S2).

**Table 1:** Assay Precision: *tbx21* positive CMV-specific CD4 T cells.

						Exact binomial 95%CI		
Sample	Cells/plate	Responses	SD	CV	% Response	62 cells	186 cells	Width of CI
S1–1	62	16	2.12	11.16	25.81	15.53	38.50	22.97
S1–2	62	18	0.71	3.72	29.03	18.20	41.95	23.75
S1–3	62	23	2.83	14.88	37.10	25.16	50.31	25.14
**S1**	**Total = 186**	**57**		**9.92**	**30.65**	**24.11**	**37.81**	**13.70**
S2–1	62	26	1.88	8.08	41.94	29.51	55.15	25.64
S2–2	62	22	0.394	4.03	35.48	23.74	48.66	24.92
S2–3	62	22	0.94	4.03	35.48	23.74	48.66	24.92
**S2**	**Total = 186**	**70**		**10.13**	**37.63**	**30.65**	**45.02**	**14.37**
S3–1	62	20	1.88	8.31	32.26	20.94	45.34	24.40
S3–2	62	24	0.94	4.15	38.71	26.60	51.93	25.33
S3–3	62	24	0.94	4.15	38.71	26.60	51.93	25.33
**S3**	**Total = 186**	**68**		**5.54**	**36.56**	**29.63**	**43.92**	**14.29**
S4–1	62	23	0.234	1.01	37.10	25.16	50.31	25.14
S4–2	62	25	1.18	5.06	40.32	28.05	53.55	25.50
S4–3	62	22	0.94	4.03	35.48	23.74	48.66	24.92
**S4**	**Total = 186**	**70**		**3.37**	**37.63**	**30.65**	**45.02**	**14.37**
			**Mean CV**	**6.05**		**Average width per plate**		**24.83**
						**Average width for 3 plates**		**14.18**

3 plates of CMV specific CD4 cells we sorted and analyzed using the scRT-PCR assay. Results for Tbet are shown. Average CV between the 3 plates for each individual were <10.13%, with the average between all 4 samples being 6.05%. The precision of the assay calculated as the width of CI for three plates is 57.1% more precise that form a single plate.

### Applications of the assay

**Figure 4:**
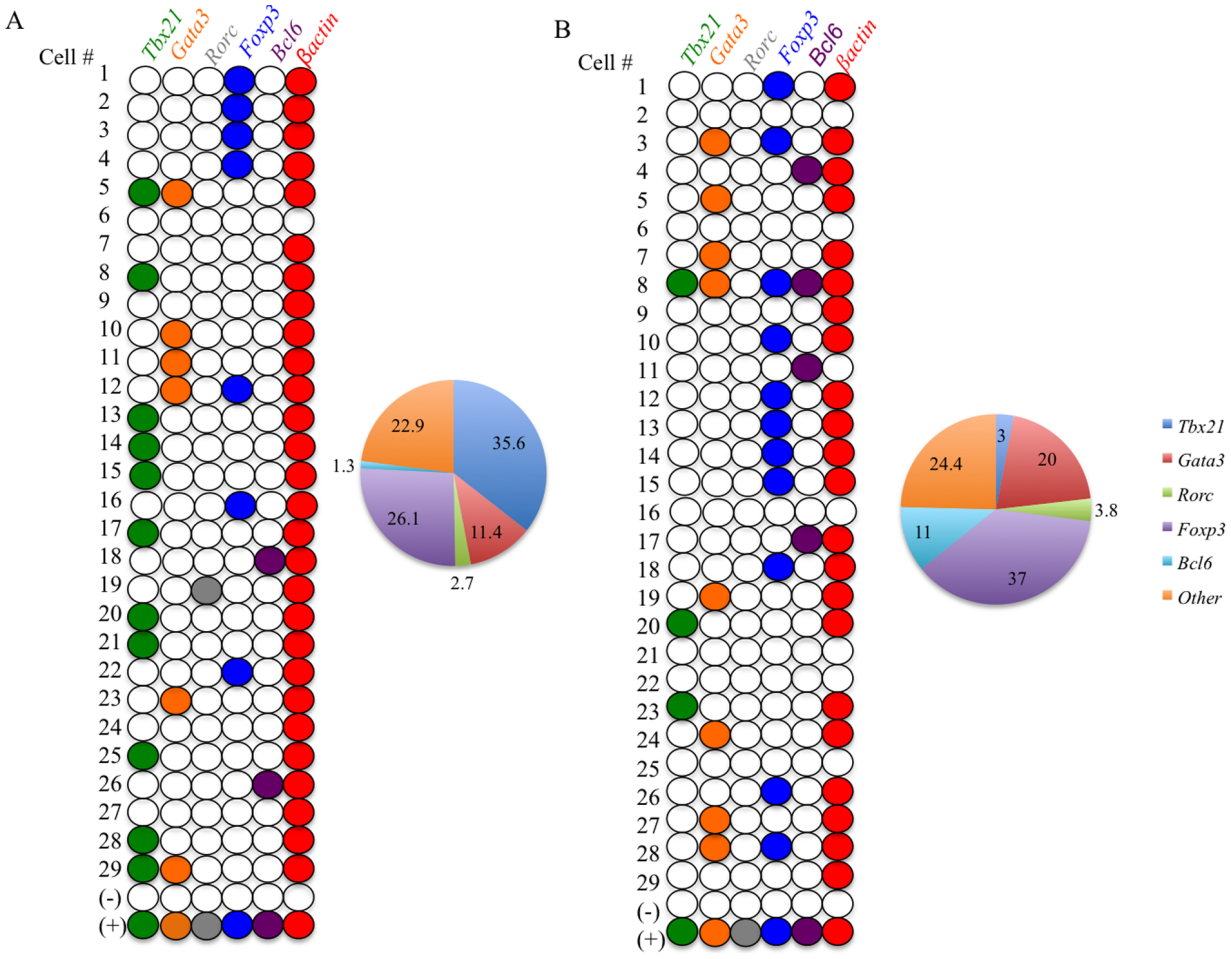
Applications of the assay. CMV specific CD4 T cells were single cell sorted and used for the scRT-PCR assay. Expression of the 5 TF and *ß-actin* was detected using real-time PCR. The numbers on the left hand side represent the cell numbers, with positive (activated CD4 T cell RNA) and negative (no cell) controls. 29 individual cells are represented here from the total of 62 cells. Colored circles represent the presence of the TF within the cell. A) CMV specific single cells (representative result of 3 experiments performed on each of 4 individuals) TF profile with Pie chart. B) TT specific single cells (representative result of 3 experiments performed on each of 2 individuals). (+) represents the positive RNA control extracted from activated CD4 T cells, (−) represents the no cell negative control. Cells that do not express *ß-actin* are excluded.

To elucidate the expression profile of Ag specific cells and to elucidate the level of heterogeneity with this population we used individually sorted Ag specific single-cells. PBMC from known CMV sero-positive healthy volunteers were used in the CD134/CD25 assay, where there was an average response of 2.26% CD25+134+ CD4+ T cells. Co-expression of CD25 and CD134 were used to define the Ag Specific population [6]. This population was then single cell sorted from 4 individuals and used for the qualitative multiplex scRT-PCR (refer to methods). Any cell that did not express *ß-actin* was classified as *‘not real’* and was excluded from further analysis. Within the CMV specific population, there were relatively high numbers of *Tbx21* expressing Th1 cells (∼35.6%) consistent with current literature suggesting that the CMV response is predominantly a Th1 type response (Fig. 4a) [21], [22]. Considerable and surprising numbers of *Foxp3* expressing T cells (∼26.1%) were found within the antigen specific population, while lower numbers of *Gata3* expressing Th2 cells (∼11.4%) were detected in this population. Tfh and Th17 cells were also present in very low numbers (∼1.3% and 2.7% respectively). A similar pattern was found for CMV responses from all 4 individuals. In addition when the same individual was tested on a separate day these ratios were stable (Table 1). A very interesting observation from these data are that they reveal the presence of only a minority of cells that co-express more than 1 lineage defining TF (e.g. *Tbx21+Gata3* and *Gata3+Foxp3*) suggesting that there maybe an extent of plasticity within these subpopulations.

Tetanus Toxoid (TT) responses were tested in 2 individuals and TT specific cells (2.82% CD25+134+ of CD4+ T cells) had a distinctly different TF expression pattern. Again ∼82% of cells sorted expressed only a single TF. There was a dominance of a Th2 type response, over Th1 responses, with 20% of cells expressing *gata3*, consistent with current literature (Fig. 4b). Similar to the CMV responses a relatively large proportion of *Foxp3* positive T cells (37%) were present and these percentages were consistent with FACS data based on the co-expression of CD39 on the cell surface [23] [24] and FoxP3 protein expression (Patent WO/2009/036521). There were small numbers of Th1 (*Tbx21*+; 3%) and Tfh (*Bcl-*6+; 11%) cells within this sample. This data set correlates well with current literature, in that a Th2 type response is found following Tetanus Toxoid immunization [25]. These data are consistent with protein expression measured within the antigen specific population, whereby CMV specific cells had a significantly higher proportion of cells expressing Tbet (18.76%) compared to Tetanus Toxoid specific cells (3.75%) (Figure S3). This is also consistent with lineage heterogeneity of the Ag specific CD4+ T cell population, where differing ratios of the different functional subsets may be correlated to the level of immunological response or disease progression. Further they reveal an unexpected level of memory Treg involved in recall responses to antigen.

## Discussion

The study of cellular heterogeneity within small populations of cells at the single cell level is a powerful tool that can be used to elucidate the complexities of systems biology. Development of single-cell gene profiling was pioneered in the early 90′s to elucidate the different cell types present in heterogeneous tissues. Different classes of neurons, as well as rare cell types such as stem cells, neoplastic cells and oocytes were the first to be targeted for single cell profiling [10], [26]–[28]. Single-cell gene profiling is now used more widely and has revealed great insight into the heterogeneity of transcript expression patterns. The complexities related to expression profiles are believed to rely on both qualitative and quantitative differences that may determine the fate of the cell. However, assays designed for the measurement of these differences still lack sensitivity and specificity [29], [30].

To better understand the heterogeneity within human Ag specific CD4+ T cells, we designed a qualitative multiplex single-cell RT-PCR assay that allows the detection of lineage determining TF expression within an individual circulating lymphocytes. The simultaneous detection of TF transcripts (up to 12 genes) in one cell, allows the identification of the specific lineage of each CD4+ T cell responding to an antigen, as well as giving insight into the plasticity of these cells when co–expression of 2 or more TF are present. The *Stats* (Signal transducers and activators of transcription) were included as target TF in order to be used as secondary criteria to further confirm the identity of each CD4+ T cell subset. However >77% of cells expressed 2 or more *Stats*, with some cells expressing all. Therefore the *Stat* profiling did not aid with the attribution of specific differentiation pathways to the single cells within these complex antigen specific populations. A caveat with using *Stats* is that they can be up regulated by signals down stream of cell surface co-receptors and cytokine receptors that will be stimulated during the 44 hr incubation period and these signals are not necessarily lineage specific.

While a quantitative assay may be useful for the description of the differences in transcript expression within one cell where gene expression can vary tremendously, the focus here is to determine the extent of qualitative heterogeneity within the Ag specific T cells, which likely correlates with the functional fate of each cell. This is based on the hypothesis that the ratios of lineage specific subsets of CD4+ T cells within the antigen specific population will vary and will impact on the outcome of that response. Thus a qualitative PCR was sufficient to answer this question. The ratios of lineage subsets can be determined by simply scoring the cells as either expressing or not expressing each of the lineage determining TFs. This is feasible because the overwhelming majority of cells (∼82%) express only one of these TFs.

A qualitative multiplex scRT-PCR assay is able to detect gene expression profiles within single cells, only when there is strict compliance to several parameters. The specificity of the amplification, sensitivity of the assay, reverse transcription efficiency, primer competition and linear dynamic range are all very important factors that have been systematically optimized and validated as these parameters are interdependent.

Previous studies on single-cell gene expression were limited to a small number of genes (∼4–5), as it was believed that amplification of greater numbers of genes would lead to PCR inhibition due to increased competition [31] [32]. More recent studies have shown that a larger number of genes (∼20–72) can be detected at a single-cell level. The critical steps in this extended multiplex assay, appears to be the designing gene specific 3′ reverse primers for the RT step and addressing primer competition to avoid PCR inhibition [11] [33]. Peixoto et al and Stanley et al both describe multiplex 2-step RT-PCR procedures that use gene specific primers for reverse transcription, and require a pre-amplification step (15–20 cycles), however both studies use SYBR®-green I dye which limits the specificity of the assays when compared with LNA hydrolysis probes.

The qualitative multiplex scRT-PCR assay uses 3′ gene-specific primers for the reverse transcription step similar to the assay described above, however lower concentrations of the 24 primers used in this assay (range: 40 nM – 25 nM) both limits residual carry over into the 2^nd^ round PCR and primer competition. Superscript III/*Taq* polymerase was used for the 1^st^ round pre-amplification where the optimal RT temperature is 51°C. The Superscript III was denatured at 95°C for 2 min and the optimal pre-amplification cycle was 22, where the assay remains linear. Higher cycle numbers within the 1^st^ round pre-amplification step decrease PCR efficiency, as reagents are used up particularly for the more prevalent transcripts and the reaction may exceed the exponential phase important for quantification in the second round real-time PCR. Specificity of the assay is achieved through the use of LNA hydrolysis probes that have superior specificity when compared to assays that use SYBR®-green dye. The scRT-PCR assay is also sensitive down to 10 fg, a log lower than previously reported assays [11] [11]. Further, this scRT-PCR has the potential for quantitative detection of genes within a single cell.

This assay was designed as a qualitative tool that can determine the identity of a cell based on the expression on lineage defining transcription factors. Ag specific CD4+ T cells are a population of cells of interest to the fields of vaccine development, immuno-virology and basic immunology. Analysis of this population has been cumbersome due to their small number found in peripheral blood and many studies have been based on cells that were first expanded by in vitro proliferation using IL-2, which almost certainly changes the nature of the cells compared to undivided cells ex vivo. FACS analysis using CD134 and CD25 co-expression reveals the presence of Ag specific cells but by itself cannot determine the actual heterogeneity of the cells within this population, that is, whether they are Th1, Th2, Th17, Tfh or Tregs or a mixture of all 5. The scRT-PCR assay allows an approach to this determination. It can give insight into the identity of each individual cell as well as elucidate the presence of plasticity and heterogeneity with these small populations. The assay reveals that the overwhelming majority of these cells express only one of these transcription factors. It also reveals unexpected and substantial heterogeneity within the antigen specific population, including surprising levels of cells expressing Foxp3 (the TF determining Treg lineage). The scRT-PCR assay has been successfully developed and validated for the qualitative detection of lineage defining TF for the elucidation of individual CD4+ T cell subsets and can be used to determine the complexities within small populations of cells, where cell numbers are limited. Importantly it may be applicable to monitoring immune responses in human tissue.

In conclusion, we describe the development and validation of a qualitative multiplex single-cell RT-PCR assay that has great specificity and sensitivity that can be used for the identification of a very minute population of cells. Further while some lineage determining transcription factors such as T-bet and FoxP3 can be reliably detected by flow cytometry, others such as Rorγt, GATA3 and Bcl-6 are more problematical. Preliminary data have revealed that a population of cells that was previously thought to be homogenous is actually a heterogeneous population. This assay has the ability to detect simultaneously the presence of multiple genes present within a single-cell and is a powerful tool that can reveal the complexities of systems biology.

## Supporting Information

Figure S1 A) Sorting 1 cell per well. PBMC surrounding 1 HUT78 cell in normal light microscopy. Fluorescent HUT78-GFP+ cell in the GFP channel, confirming that FACS Aria reproducibly sorts 1 cell per well. B) Sensitivity of the assay was analyzed by titrating total RNAextracted from PMA/Ionomycin activated CD4+ T cells; from 100 pg to 10 fg. Most of the TF were detectable down to 10 fg, except for *Roryt*, which was present at much lower in copy numbers but were still detectable at 100 fg of total cellular RNA.
(TIF)

Figure S2 Elucidating pre-amplification cycle number. A) *Bcl-6* plasmid standards (10^7^–10 copies) were used in the 1^st^ round pre-amplification step with 22 or 30 cycles of pre-amplification. At 22 cycles the 1^st^ round assay remains linear with an r^2^ = 0.996, however at 30 cycles the standard curve is no longer linear with an r^2^ = 0.869. B) Differing input RNA concentrations were used at 22 cycles (1ng, 100 pg and 10 pg). Transcription factor transcripts were detectable at all concentrations of input RNA.
(TIF)

Figure S3 Tbet and Foxp3 protein expression in Antigen specific subset. A) Flow cytometry dot plot showing Tbet vs. Foxp3 expression gated from CMV specific CD4+CD25+Ox40+ T cells. B) Flow cytometry dot plot showing Tbet vs. Foxp3 expression gated from Tetanus toxoid specific CD4+CD25+Ox40+ T cells. C) Tbet and Foxp3 expression in CMV and Tetanus toxoid specific cells from 8 individuals. Wilcoxon paired *t* test was used to calculate significance, *P* value less than 0.05 was considered significant.
(TIF)

Table S1 Primer Design. A) 12 primer sets for lineage determining transcription factors (TF) and STATs were designed using **ProbeFinder** software. Primers range from 18–28 bp with approximately 50% GC content and Tm <60°C. Secondary structure or Hairpins were excluded if their ΔG is lower than −3 kcal/mol and Tm >50°C. Hetero-dimer of the primer to its target template less than −30 kcal/mol indicates spontaneous interaction. B) UPL® LNA probes with company catalogue numbers.
(TIF)

Table S2 Assay Precision: *Gata3* positive TT-specific CD4 T cells. 3 plates of TT-specific CD4 cells we sorted and analyzed using the scRT-PCR assay. Average CV between the 3 plates for each individual were <15.25%, with the average between all 3 samples being 10.78%. The precision of the assay calculated as the width of CI for three plates is 57.6% more precise than from a single plate.
(TIF)
