# Supplementary figures and images for: Characterization of Transcription Factor Phenotypes within Antigen-Specific CD4+ T Cells Using Qualitative Multiplex Single-Cell RT-PCR
Source: PLoS One. 2013 Oct 4;8(10):e74946. doi: 10.1371/journal.pone.0074946 (PMC3790772; doi:10.1371/journal.pone.0074946)

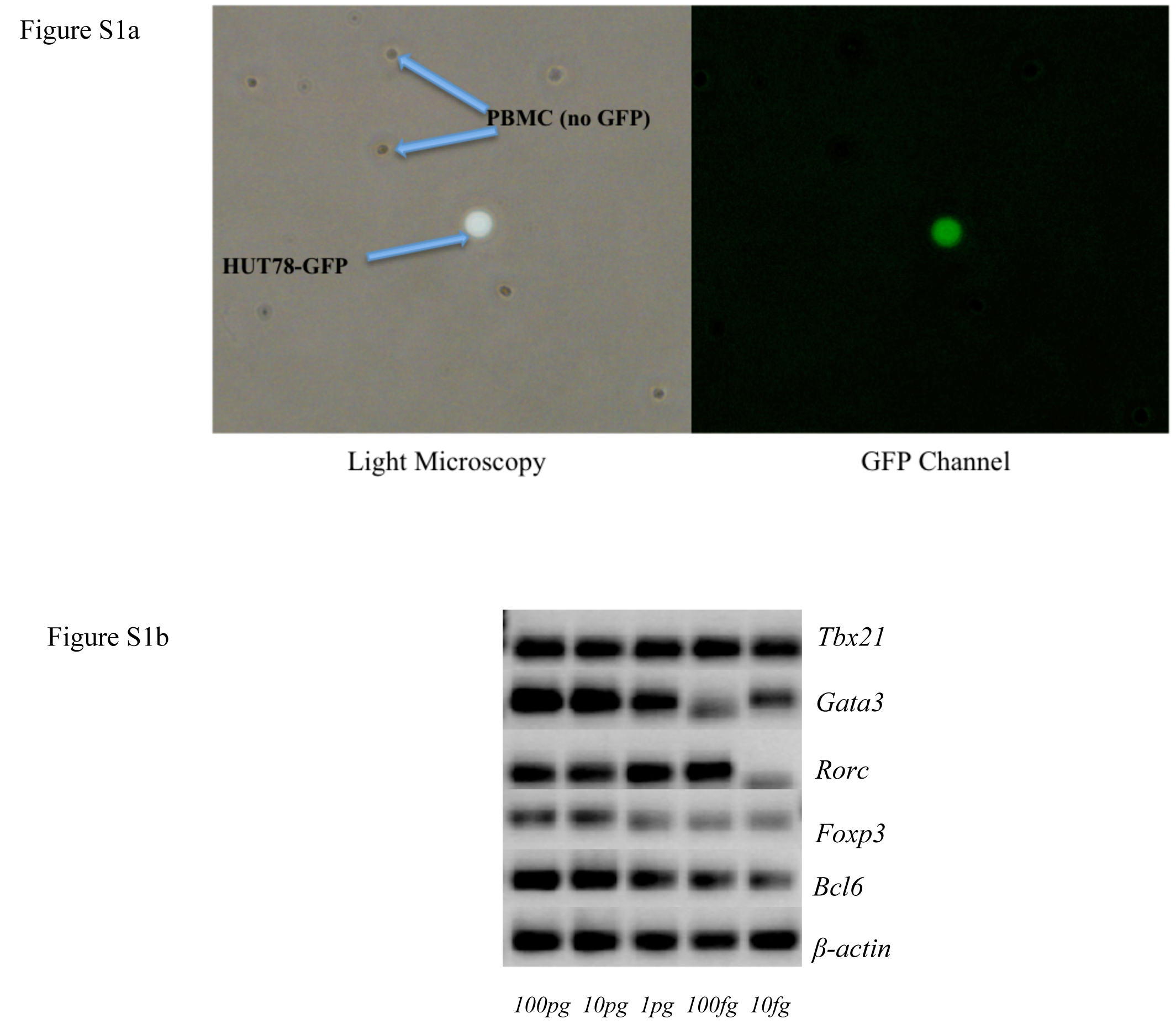

Supplement: Figure S1 — A) Sorting 1 cell per well. PBMC surrounding 1 HUT78 cell in normal light microscopy. Fluorescent HUT78-GFP+ cell in the GFP channel, confirming that FACS Aria reproducibly sorts 1 cell per well. B) Sensitivity of the assay was analyzed by titrating total RNAextracted from PMA/Ionomycin activated CD4+ T cells; from 100 pg to 10 fg. Most of the TF were detectable down to 10 fg, except for Roryt, which was present at much lower in copy numbers but were still detectable at 100 fg of total cellular RNA. (TIF) [file pone.0074946.s001.tif]

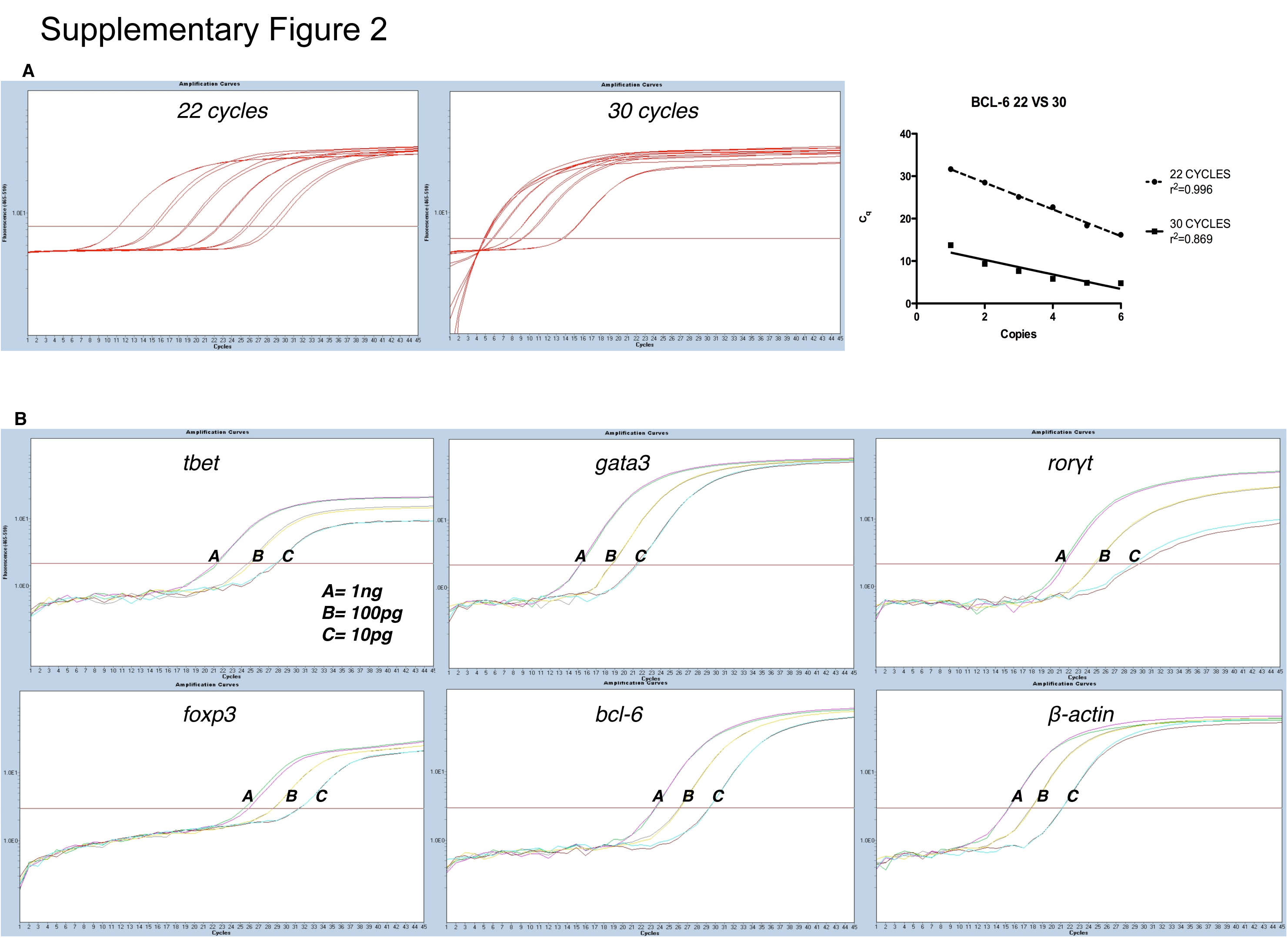

Supplement: Figure S2 — Elucidating pre-amplification cycle number. A) Bcl-6 plasmid standards (107–10 copies) were used in the 1st round pre-amplification step with 22 or 30 cycles of pre-amplification. At 22 cycles the 1st round assay remains linear with an r2 = 0.996, however at 30 cycles the standard curve is no longer linear with an r2 = 0.869. B) Differing input RNA concentrations were used at 22 cycles (1ng, 100 pg and 10 pg). Transcription factor transcripts were detectable at all concentrations of input RNA. (TIF) [file pone.0074946.s002.tif]

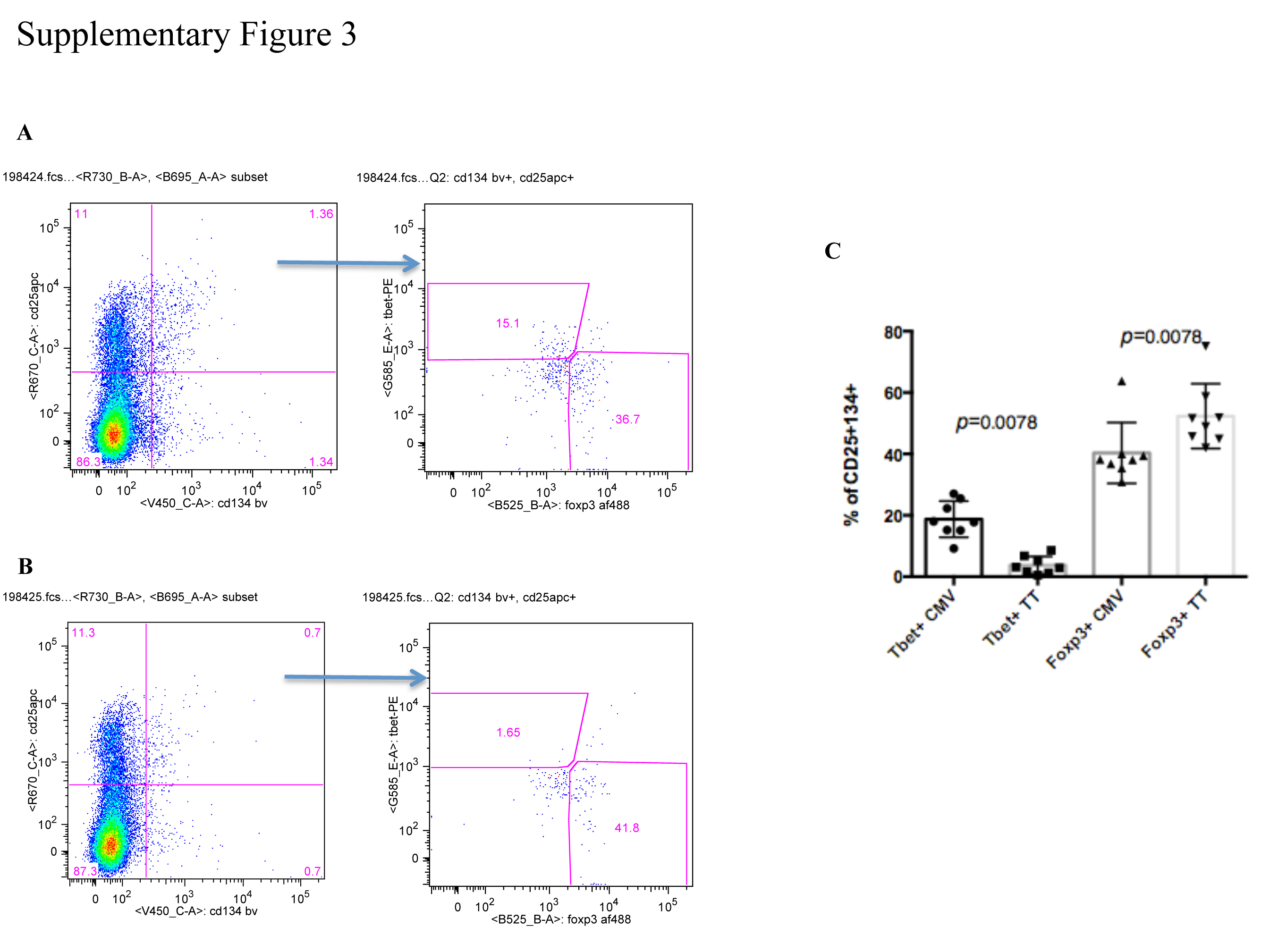

Supplement: Figure S3 — Tbet and Foxp3 protein expression in Antigen specific subset. A) Flow cytometry dot plot showing Tbet vs. Foxp3 expression gated from CMV specific CD4+CD25+Ox40+ T cells. B) Flow cytometry dot plot showing Tbet vs. Foxp3 expression gated from Tetanus toxoid specific CD4+CD25+Ox40+ T cells. C) Tbet and Foxp3 expression in CMV and Tetanus toxoid specific cells from 8 individuals. Wilcoxon paired t test was used to calculate significance, P value less than 0.05 was considered significant. (TIF) [file pone.0074946.s003.tif]

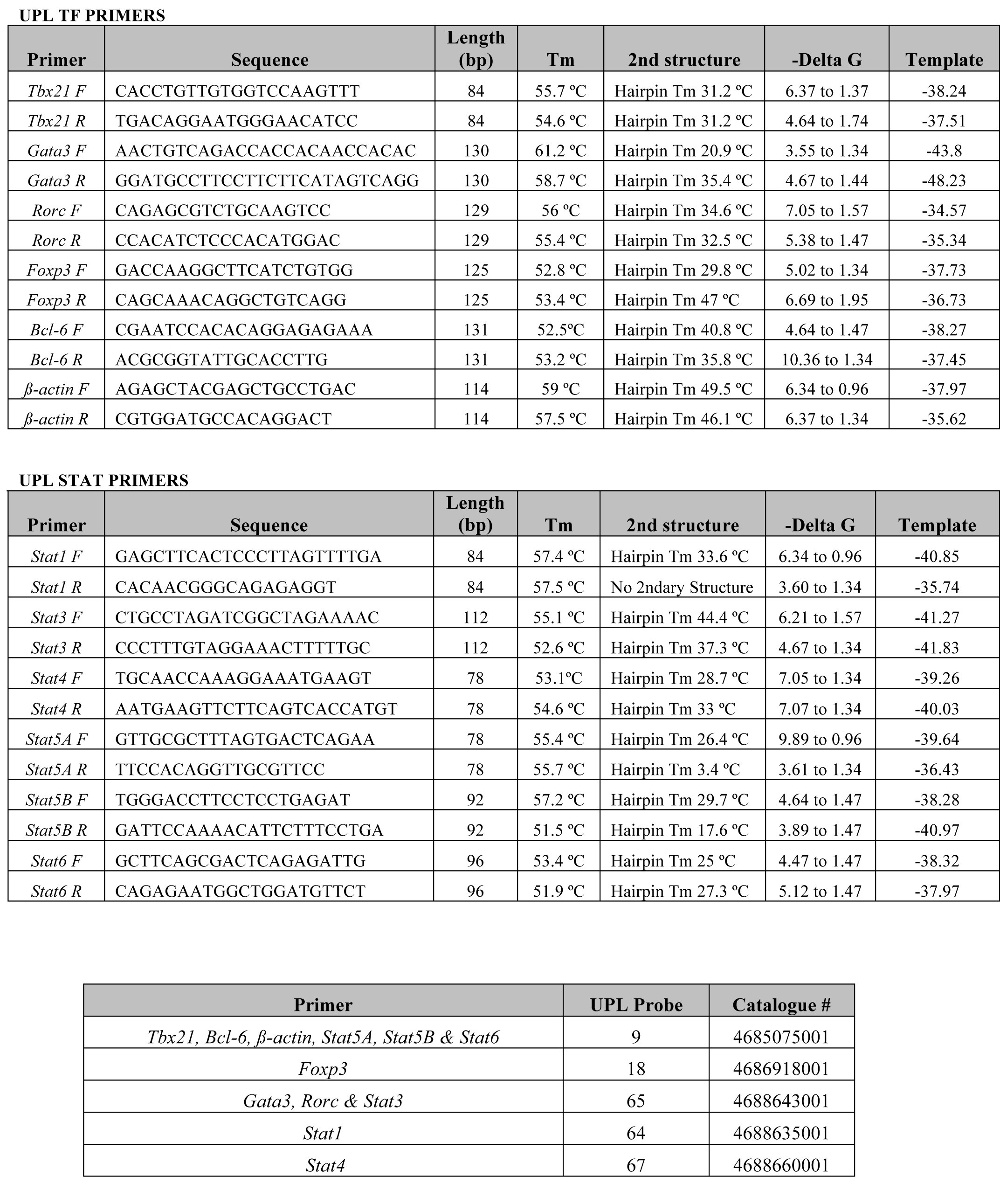

Supplement: Table S1 — Primer Design. A) 12 primer sets for lineage determining transcription factors (TF) and STATs were designed using ProbeFinder software. Primers range from 18–28 bp with approximately 50% GC content and Tm <60°C. Secondary structure or Hairpins were excluded if their ΔG is lower than −3 kcal/mol and Tm >50°C. Hetero-dimer of the primer to its target template less than −30 kcal/mol indicates spontaneous interaction. B) UPL® LNA probes with company catalogue numbers. (TIF) [file pone.0074946.s004.tif]

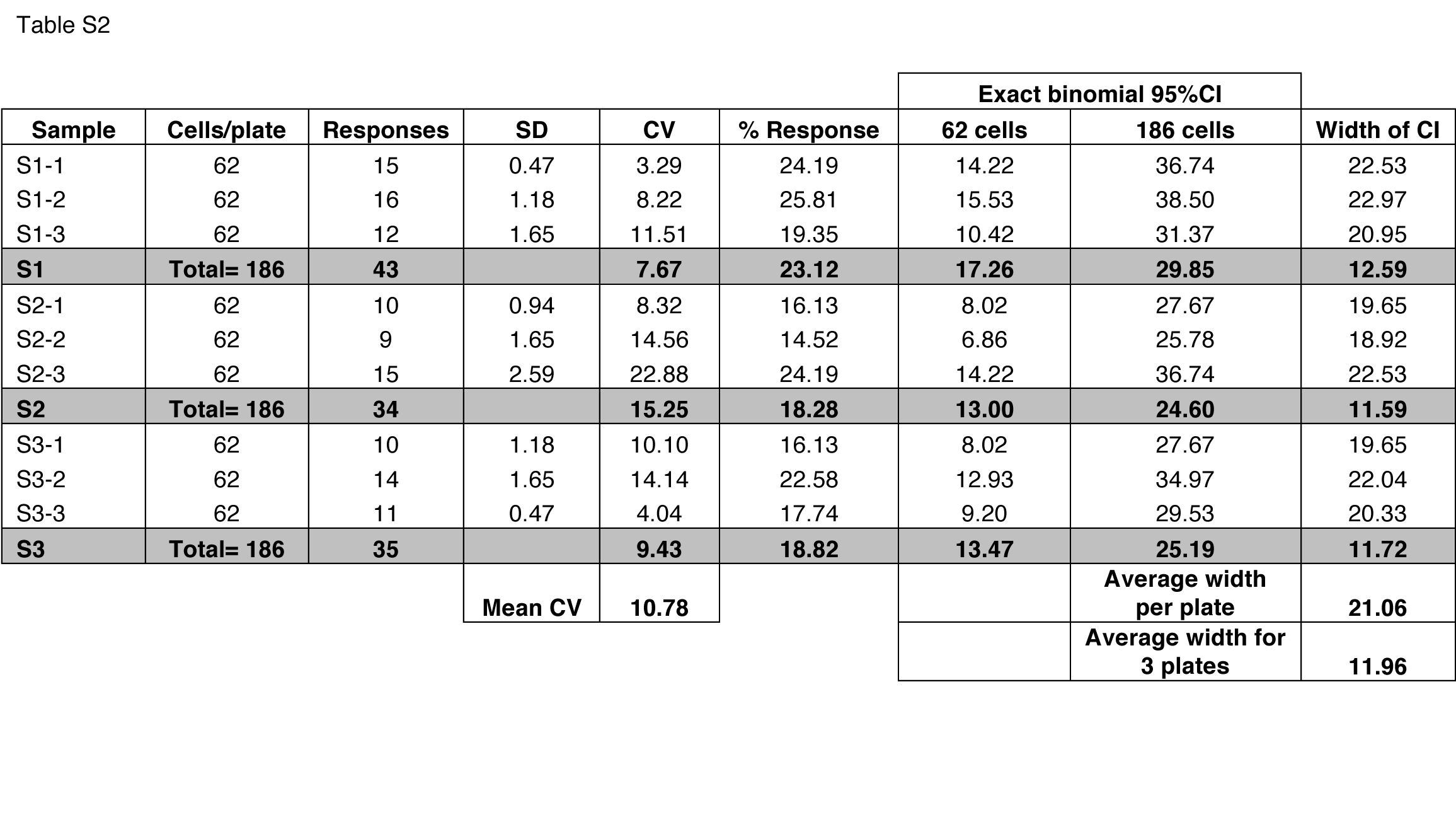

Supplement: Table S2 — Assay Precision: Gata3 positive TT-specific CD4 T cells. 3 plates of TT-specific CD4 cells we sorted and analyzed using the scRT-PCR assay. Average CV between the 3 plates for each individual were <15.25%, with the average between all 3 samples being 10.78%. The precision of the assay calculated as the width of CI for three plates is 57.6% more precise than from a single plate. (TIF) [file pone.0074946.s005.tif]
